# Effects of strain rate on room- and cryogenic-temperature compressive properties in metastable V10Cr10Fe45Co35 high-entropy alloy

**DOI:** 10.1038/s41598-019-42704-x

**Published:** 2019-04-16

**Authors:** Hyejin Song, Dong Geun Kim, Dae Woong Kim, Min Cheol Jo, Yong Hee Jo, Wooyeol Kim, Hyoung Seop Kim, Byeong-Joo Lee, Sunghak Lee

**Affiliations:** 1 Center for High Entropy Alloys, Pohang University of Science and Technology, Pohang, 790-784 Korea; 2 Extreme Fabrication Technology Group, Korea Institute of Industrial Technology, Daegu, 42994 Korea

## Abstract

Quasi-static and dynamic compressive properties of an FCC-based metastable HEA (composition; V10Cr10Fe45Co35 (at.%)) showing both Transformation Induced Plasticity (TRIP) and TWinning Induced Plasticity (TWIP) were investigated at room and cryogenic temperatures. During the quasi-static and dynamic compression at room temperature, the FCC to BCC TRIP occurred inside FCC grains, and resulted in very high strain-hardening rate and consequently maximum compressive strength over 1.6 GPa. The dynamic compressive strength was higher by 240 MPa than the quasi-static strength because of strain-rate-hardening effect, and kept increasing with a high strain-hardening rate as the twinning became activated. The cryogenic-temperature strength was higher than the room-temperature strength as the FCC to BCC TRIP amount increased by the decrease in stability of FCC phase with decreasing temperature. Under dynamic loading at cryogenic temperature, twins were not formed because the increase in SFE due to adiabatic heating might not be enough to reach the TWIP regime. However, the dynamically compressed specimen showed the higher strength than the quasi-statically compressed specimen as the strain-rate-hardening effect was added with the TRIP.

## Introduction

Preservation, development, and transportation of resources have demanded novel metal alloys having excellent mechanical properties for technological advances and applications to extreme environments. Though unceasing efforts have been made to enhance mechanical properties, cryogenic-temperature environments often cause a severe deterioration in ductility and toughness by lowering damage-tolerance capacities^1,2^. Recently-developed face-centered-cubic(FCC)-based high-entropy alloys (HEAs) provide excellent cryogenic-temperature strength, ductility, and fracture toughness by actively generating a TWinning Induced Plasticity (TWIP) mechanism^3–6^. So as to further enhance mechanical properties at cryogenic temperature, various strengthening mechanisms including solid solution hardening^7^, precipitation hardening^8^, and grain refinement^9,10^ have been suggested.

Recently, a TRansformation Induced Plasticity (TRIP) mechanism widely utilized in austenitic high-Mn steels has been adopted to HEAs^11,12^. For example, Z. Li *et al*. found an FCC to hexagonal-close-packed (HCP) TRIP in FeMnCoCr or CoCrFeMnNi alloys^9,11,13,14^. In austenitic high-Mn steels, both TWIP and TRIP are working, depending on their stacking fault energy (SFE), and the TRIP becomes more active as the SFE goes down with decreasing temperature. Though the definition and utilization of SFE are difficult in FCC-based HEAs, the transition in deformation mechanisms might occur with decreasing temperature, and can directly affect their cryogenic-temperature properties^15^.

However, the dynamic deformation behavior is hardly investigated because most of HEA researches deal with static or quasi-static mechanical phenomena. Applied strain rates often change deformation mechanisms, and the hardening occurs under dynamic strain rates as the thermal activation of dislocation movement is often restricted by a viscous drag effect^16,17^. An adiabatic heating also occurs because the heat generated during the dynamic deformation is not sufficiently emitted. TRIP and TWIP mechanisms can be activated by this temperature rise due to adiabatic heating, but which mechanism critically affects dynamic properties has not been elucidated. Thus, dynamic deformation researches are essentially needed for cryogenic or extreme applications of HEAs to enhance quasi-static and dynamic mechanical properties and to understand dynamic deformation behavior.

Quasi-static and dynamic compressive properties of an FCC-based metastable HEA showing both TRIP and TWIP were investigated at room and cryogenic temperatures by using a universal testing machine and a split Hopkinson pressure bar, respectively, in this study. A transition phenomenon of TRIP and TWIP mechanisms occurring under different loading conditions and test temperatures was also examined.

## Results

### HEA microstructure

The achievement of a single FCC phase in an annealed state and the increase in thermal stability of BCC phase at low temperatures are used to generate the FCC to BCC martensitic transformation in the present HEA design. Based on the CALPHAD approach with a Thermo-Calc software^18^, Co, Mn, and Ni contents are varied in the V_10_Cr_10_Fe_45_Co_x_Mn_(35−x)_ and V_10_Cr_10_Fe_45_Co_x_Ni_(35−x)_ systems, whose equilibrium phase diagrams at 600 °C~1500 °C are shown in Supplementary Fig. 1a,b. Supplementary Fig. 1c,d shows differences in Gibbs free energies between BCC and FCC phases (ΔG^FCC→BCC^, stability of BCC phase) at 25 °C. The lower ΔG^FCC→BCC^ indicates the high stability of BCC phase. The stability of BCC phase increases largely with increasing Co content, as indicated by blue arrow bands in Supplementary Fig. 1c,d, irrespective of Mn or Ni content, while the stability of FCC phase decreases. In order to obtain the stable single FCC phase at 950 °C (HEA annealing temperature, dotted lines in Supplementary Fig. 1a,b) and to generate the maximal transformation from FCC to BCC, the Co content is set to be 35 at.% (HEA composition; V10Cr10Fe45Co35 (at.%)) in this study.

Supplementary Fig. 2a,b shows an EBSD phase map and XRD data of the V10Cr10Fe45Co35 HEA. The HEA shows a single phase of FCC (Supplementary Fig. 2a), and its average size of FCC grains is 20 μm. XRD peaks (Supplementary Fig. 2b) show the same results with the phase map, which satisfies the first objective of the present HEA design.

### Quasi-static and dynamic compressive properties at room temperature

**Figure 1:**
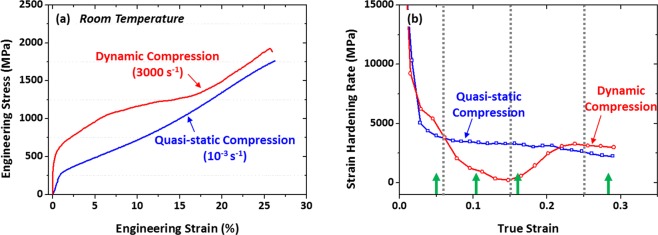
Representative quasi-static and dynamic (**a**) engineering stress-strain curves and (**b**) strain-hardening-rate (dσ/dε) curves tested at room temperature of the V10Cr10Fe45Co35 HEA. The dynamic compressive specimen was not fractured, and thus the quasi-static compressive test was conducted until the total strain reaches 26.2% (similar to the dynamic total strain (25.9%). The dynamic yield and maximum strengths are higher than the quasi-static ones.

Figure 1a shows representative quasi-static and dynamic engineering stress-strain curves tested at room temperature, and measured compressive properties are listed in Supplementary Table 1. The dynamic compressive specimen was not fractured, and the yield compressive strength, maximum strength, and total strain are 640 MPa, 1925 MPa, and 25.9%, respectively. The quasi-static compressive test was conducted until the total strain reaches 26.2% (similar to the dynamic total strain (25.9%). The dynamic yield and maximum strengths are higher than the quasi-static ones according to a typical strain-rate hardening effect^19,20^.

Figure 1b shows strain-hardening-rate (dσ/dε) curves tested at room temperature. The quasi-static hardening rate rapidly decreases until the true strain of 0.03, and then decreases very slowly. Under dynamic compression, the strain-hardening rate decreases and then increases from the strain of 0.15. The quasi-static hardening rate is higher in the strain range of 0.06~0.22 than the dynamic hardening rate.

### Compressive deformation mechanisms at room temperature

**Figure 2:**
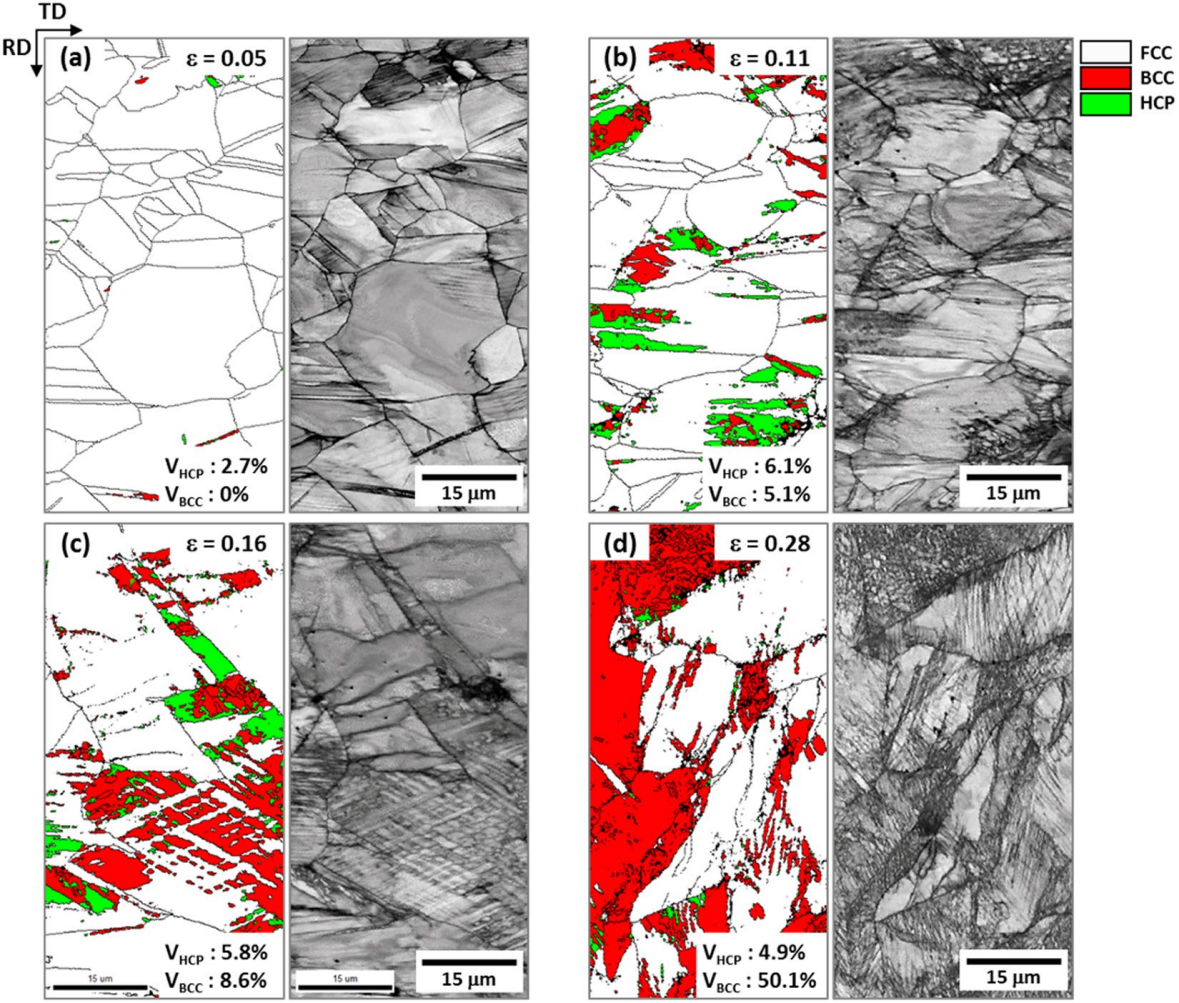
EBSD phase and image quality (IQ) maps of the cross-sectional area of the quasi-statically compressed specimen at true strains of (**a**) 0.05, (**b**) 0.11, (**c**) 0.16, and (**d**) 0.28 (green arrow marks in Fig. 1(b)). The volume fractions of HCP and BCC phases (V_HCP_ and V_BCC_, respectively) were measured, and are shown inside phase maps. At the strain of 0.11, considerable amounts of HCP and BCC phases are observed, while deformation twins are not found. Some HCP phases are independently formed, whereas all BCC phases are formed with HCP phases, which implies that most of BCC phases are transformed from already-formed HCP phases.

Figure 2a–d shows EBSD phase and image quality (IQ) maps of the cross-sectional area of the quasi-statically compressed specimen at true strains of 0.05, 0.11, 0.16, and 0.28 (green arrow marks in Fig. 1b). The volume fractions of HCP and BCC phases (V_HCP_ and V_BCC_, respectively) were measured, and are shown inside phase maps. At the strain of 0.05, very few HCP and BCC phases start to form at annealing twins or FCC grain boundaries (Fig. 2a). This implies that the HEA deforms mostly by the dislocation slip, rather than the TRIP or TWIP. At the higher strain (0.11), considerable amounts of HCP and BCC phases are observed, while deformation twins are not found (Fig. 2b). These HCP and BCC phases are martensites transformed from the FCC phase by the compressive deformation. Some HCP phases are independently formed, whereas all BCC phases are formed with HCP phases, which implies that most of BCC phases are transformed from already-formed HCP phases. With the further compression (strain; 0.16), the formation of BCC phase becomes active as most of HCP phases transform to BCC phases (Fig. 2c). In the final deformation stage (strain; 0.28), BCC phases are quite populated, and their volume fraction reaches 50.1%, whereas only a few HCP phases are observed (Fig. 2d).

**Figure 3:**
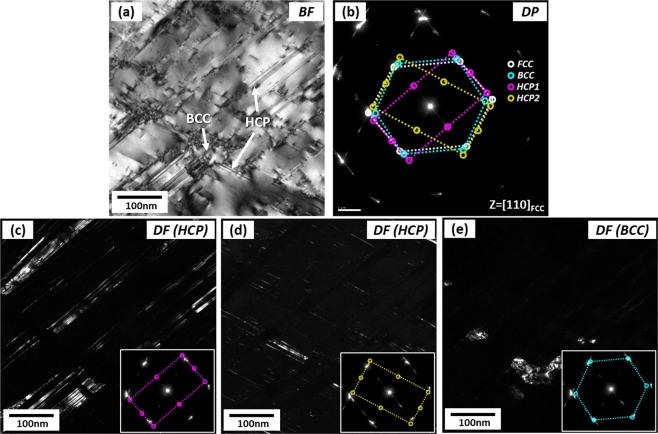
TEM (**a**) bright-field (BF) image, (**b**) selected area diffraction (SAD) patterns, and (**c**–**e**) dark-field (DF) images of parallel lines existed inside FCC grains of the quasi-statically compressed specimen at the true strain of 0.11 (Fig. 2(b)). Parallel lines are identified to be HCP phases by the SAD pattern and DF images. BCC phases are also found mostly at intersections of two HCP phases, which confirms that BCC phases are formed by the transformation from already-formed HCP phases.

In order to identify parallel lines existed inside FCC grains of the quasi-statically compressed specimen at the true strain of 0.11 (Fig. 2b) which might be HCP phases or deformation twins, TEM analyses were performed. Figure 3a–e shows TEM bright-field (BF) and dark-field (DF) images and selected area diffraction (SAD) patterns obtained from a zone axis of [−110]_FCC_//[11–20]_HCP_ . A number of parallel lines are observed inside an FCC grain (Fig. 3a), and are identified to be HCP phases by the SAD pattern (Fig. 3b) and DF images (Fig. 3c,d). BCC phases are also found at intersections of two HCP phases (Fig. 3e), which confirms again that BCC phases are formed by the transformation from already-formed HCP phases. When HCP phases composed of two kinds of parallel lines are formed from the FCC matrix, the orientation relationships between FCC and two HCP phases are (111)_FCC_//(0002)_HCP1_ and (11–1)_FCC_//(0002)_HCP2_, respectively, as shown in Fig. 3b–d.

**Figure 4:**
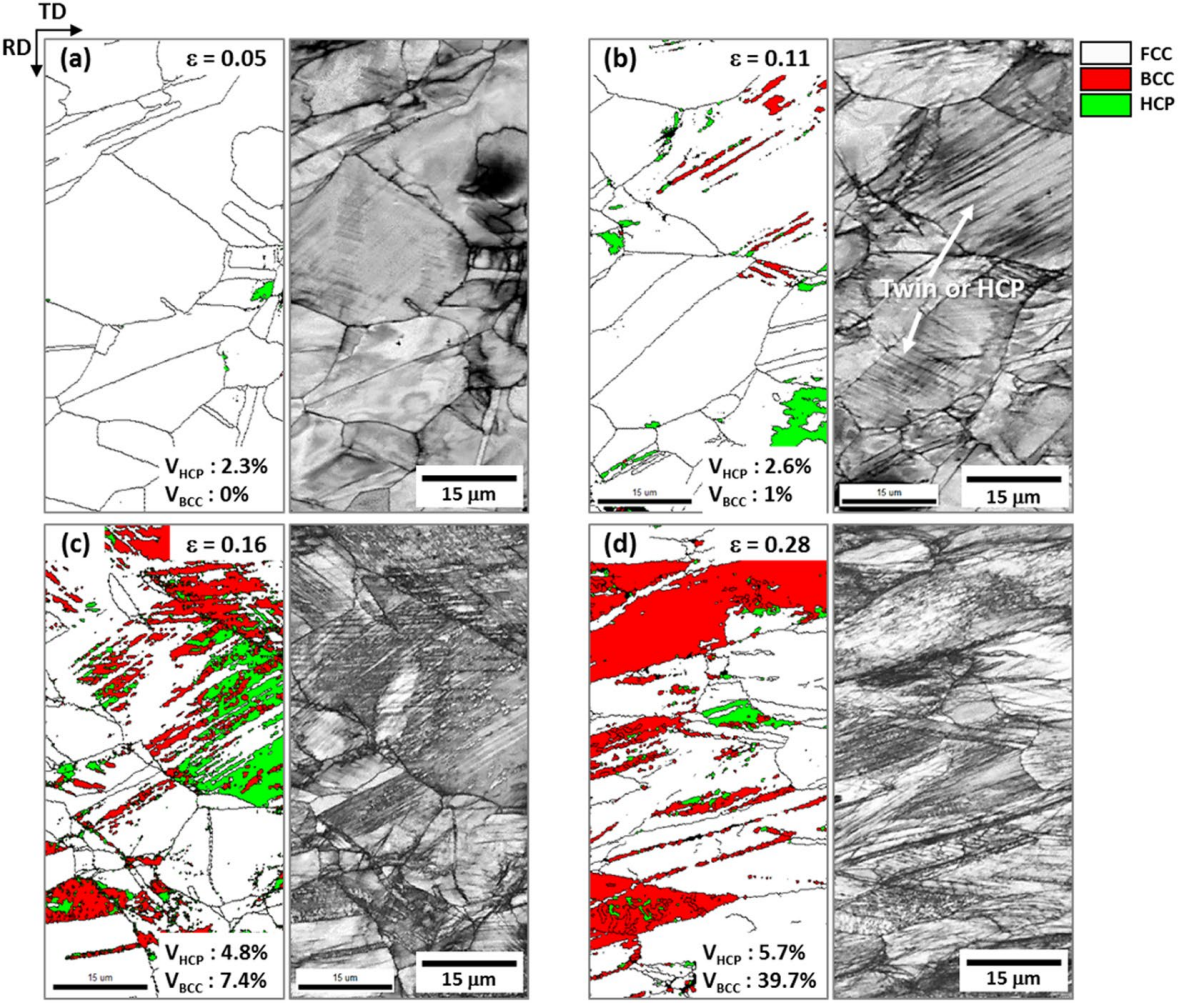
EBSD phase and IQ maps of the cross-sectional area of the dynamically compressed specimen at true strains of (**a**) 0.05, (**b**) 0.11, (**c**) 0.16, and (**d**) 0.28 (green arrow marks in Fig. 1(b)). At the strain of 0.11, parallel lines are formed, although it is not clear whether they are HCP phases or deformation twins. Along these lines, a few BCC phases are formed, while some HCP phases are formed and grown at FCC grain boundaries. At the higher strains, many BCC phases are formed at already-formed HCP phases or along parallel lines developed inside FCC grains.

Figure 4a–d shows EBSD phase and IQ maps of the specimen dynamically compressed after a stopper was inserted between Hopkinson bars to obtain sequential dynamic true strains of 0.05, 0.11, 0.16, and 0.28^19^ (green-arrow marks in Fig. 1b), like the quasi-statically compressed specimen. In the initial deformation stage (true strain; 0.05), only a few HCP phases are formed inside FCC grains (Fig. 4a). At the strain of 0.11, a considerable number of parallel lines are formed, although it is not clear whether they are HCP phases or deformation twins (Fig. 4b). Along these lines, a few BCC phases are formed, while some HCP phases are formed and grown at FCC grain boundaries. At the higher strain (0.16), a number of BCC phases are formed at already-formed HCP phases or along parallel lines developed inside FCC grains (Fig. 4c). As the compression proceeds to the strain of 0.28, BCC phases are largely grown, and parallel lines are more actively formed, whereas the volume fraction of HCP phase almost remains (Fig. 4d).

**Figure 5:**
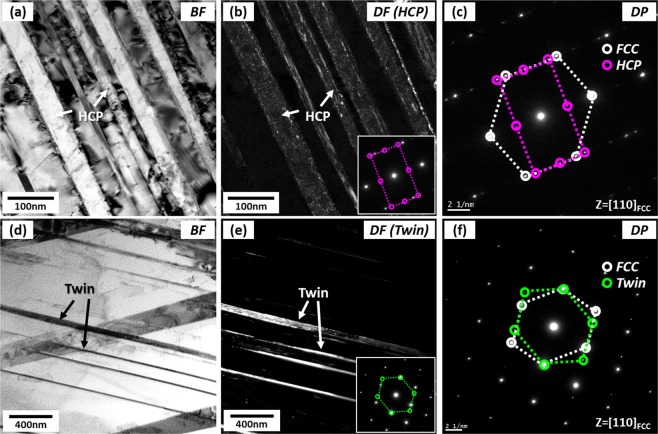
TEM (**a**,**d**) BF images, (**b**,**e**) DF images, and (**c**,**f**) SAD patterns of parallel lines existed inside FCC grains of the dynamically compressed specimen at the true strain of 0.11 (Fig. 4(b)). Somewhat thick parallel lines inside an FCC grain are identified to be HCP phases by BF and DF images and SAD pattern. In another FCC grain, thin parallel lines (several tens of nanometers) are identified to be twins.

Figure 5a–f shows TEM BF and DF images and SAD patterns of parallel lines existed inside FCC grains of the dynamically compressed specimen at the true strain of 0.11 (Fig. 4b). Somewhat thick parallel lines are identified to be HCP phases by BF and DF images (Fig. 5a,b) and SAD pattern (Fig. 5c). In another FCC grain, thin parallel lines (thickness; several tens of nanometers) are found, and are identified to be twins (Fig. 5d–f). These TEM results indicate that twins are not differentiated from HCP phases in the EBSD IQ map (Fig. 4b) because they are very thinly formed even in the TEM images. Thus, twins are formed in the dynamically compressed specimen, whereas they are not in the quasi-statically compressed specimen (Fig. 3a–e), although the quantification of their fraction is difficult.

**Figure 6:**
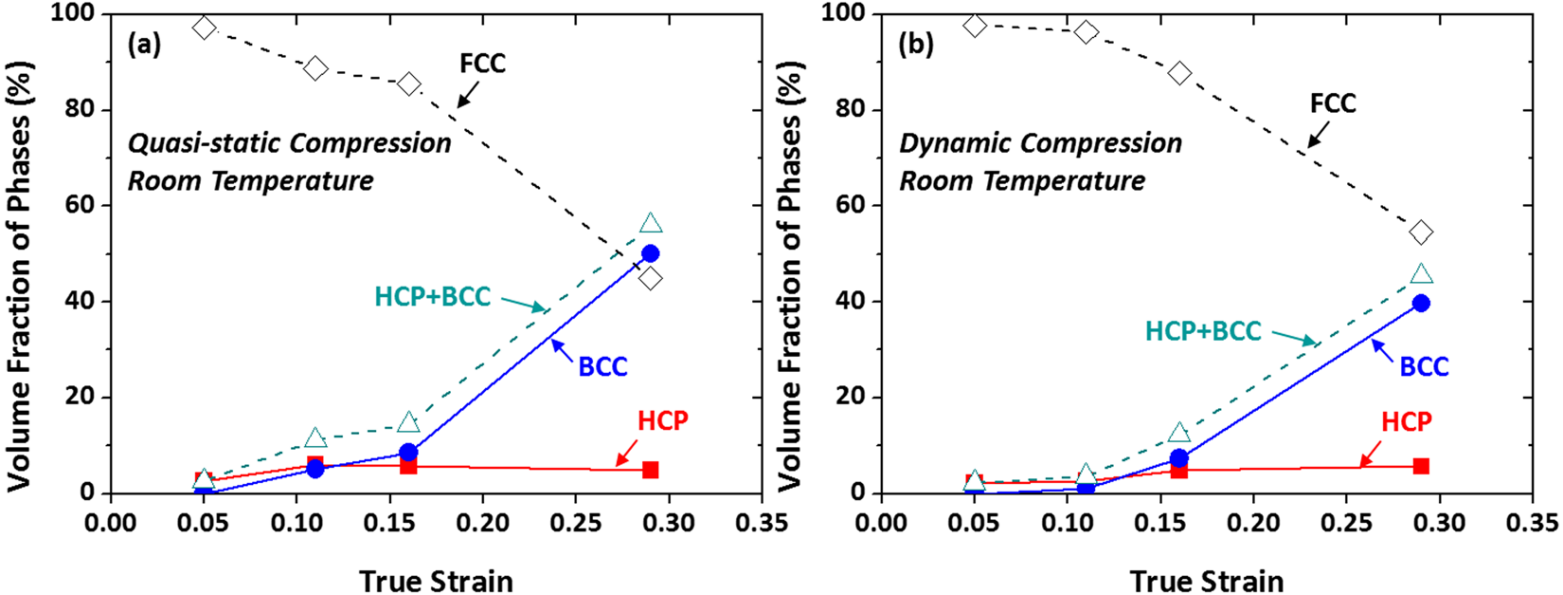
Volume fractions of FCC, BCC, and HCP phases present in the (**a**) quasi-statically and (**b**) dynamically compressed specimens as a function of compressive true strain at room temperature. Under quasi-static compression, the volume fraction of HCP phase increases to 6.1% at the strain of 0.11, and then slightly decreases until 0.28. The BCC phase is not formed at 0.05, but its fraction increases continuously to 50% with increasing strain. Trends of volume fractions of BCC and HCP phases in the dynamically compressed specimen are same to those of the quasi-statically compressed specimen, although overall volume fractions are somewhat lower in the dynamically compressed specimen.

Figure 6a,b shows volume fractions of FCC, BCC, and HCP phases present in the quasi-statically and dynamically compressed specimens as a function of true strain. Here, these fractions were measured from the XRD method^22^ described in the Experimental part because the XRD method provides the more reliable data covering the wider analysis area than the EBSD method. Under quasi-static compression, the FCC phase starts to transform to the HCP phase at 0.05 (Fig. 6a). Its fraction increases to 6.1% at 0.11, and then slightly decreases until 0.28. The BCC phase is not formed at 0.05, but its fraction increases continuously to 50% with increasing strain. The volume fraction of total (BCC + HCP) phases is governed mainly by that of BCC phase, when considering relatively low volume fractions of HCP phase. Trends of volume fractions of BCC and HCP phases in the dynamically compressed specimen are same to those of the quasi-statically compressed specimen (Fig. 6b), although overall volume fractions are somewhat lower in the dynamically compressed specimen.

### Quasi-static and dynamic compressive properties at cryogenic temperature

**Figure 7:**
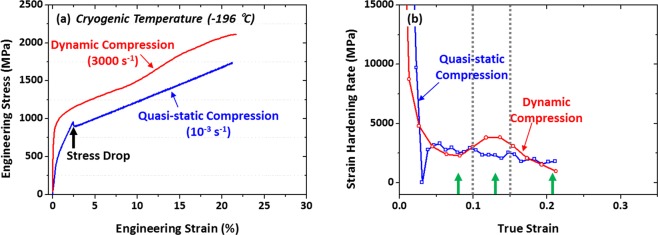
Representative quasi-static and dynamic (**a**) engineering stress-strain curves and (**b**) strain-hardening-rate (dσ/dε) curves tested at cryogenic temperature of the V10Cr10Fe45Co35 HEA. The quasi-static compressive test was conducted until the similar total strain (21.2%) to that of the dynamic compressive test. The dynamic yield and maximum strengths are higher than the quasi-static ones, and a yield-point phenomenon appears in the dynamic stress-strain curve (black arrow mark).

Figure 7a shows quasi-static and dynamic engineering stress-strain curves tested at cryogenic temperature, and measured compressive properties are shown in Supplementary Table 1. Under dynamic compression, the yield compressive strength, maximum strength, and total strain are 1083 MPa, 2245 MPa, and 21.6%, respectively. The quasi-static compressive test was conducted until the similar total strain (21.2%) to that of the dynamic compressive test (21.6%). The yield and maximum strengths of the quasi-statically compressed specimen are 534 MPa and 1735 MPa, respectively, which are lower than those of the dynamically compressed specimen. A small stress drop appears in the quasi-static stress-strain curve at a low strain level, as indicated by a black arrow. This might also appear in the dynamic stress-strain curve because it can be removed by a smoothening process of raw wave-form curves, although it is not shown in Fig. 7a. Examples of dynamic stress-strain curves are shown in Fig. 7c,d. There might exist a stress drop or stay at a low strain level, but is smoothened to produce the adjacent-averaged curve.

Figure 7b shows cryogenic-temperature strain-hardening-rate curves. The quasi-static hardening rate decreases rapidly with increasing true strain, and then decreases slowly after the true strain of 0.03, while there exists a deep well caused from the small stress drop. Under dynamic compression, it shows a down-up-down behavior, although the overall hardening rate is similar to that of the quasi-static compression.

### Compressive deformation mechanisms at cryogenic temperature

**Figure 8:**
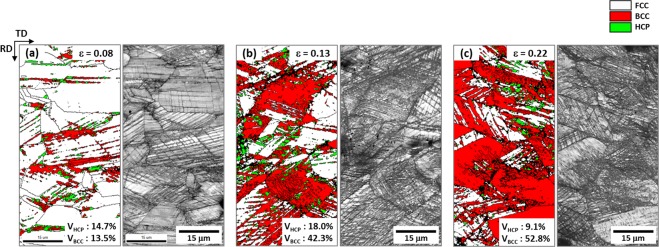
EBSD phase and IQ maps of the cross-sectional area of the dynamically compressed specimen at true strains of (**a**) 0.08, (**b**) 0.13, and (**c**) 0.22 (green arrow marks in Fig. 7(b)). At the strain of 0.08, a considerable number of HCP and BCC phases are formed along many parallel lines. Since some HCP phases are independently formed, whereas all BCC phases are formed with HCP phases.

Figure 8a–c shows EBSD phase and image quality (IQ) maps of the quasi-statically compressed specimen at true strains of 0.08, 0.13, and 0.22 (green arrow marks in Fig. 7b). At the strain of 0.08, a considerable number of HCP and BCC phases are formed along many parallel lines (Fig. 8a). Since some HCP phases are independently formed, whereas all BCC phases are formed with HCP phases, it is expected that most of BCC phases are formed in a parallel-line shape by the transformation from already-formed HCP phases. At the higher strain (0.13), a number of BCC phases are formed, while the amount of HCP phase is not varied much (Fig. 8b). As the formation of BCC phase becomes more active, most of parallel lines are crossed each other. With the further compression (strain; 0.22), most of FCC grains are filled with BCC phases together with a few HCP phases (Fig. 8c).

**Figure 9:**
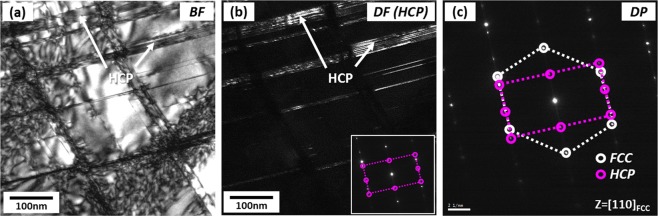
TEM (**a**) BF and (**b**) DF images and (**c**) SAD pattern of parallel lines existed inside an FCC grain of the quasi-statically compressed specimen at the true strain of 0.13. Many crossed parallel lines inside the FCC grain are identified to be HCP phases by the DF image and SAD pattern. Twins are not found in this specimen.

Figure 9a–c shows TEM images and diffraction pattern of parallel lines existed inside an FCC grain of the quasi-statically compressed specimen at 0.13 (Fig. 8b). There are many crossed parallel lines inside the FCC grain (Fig. 9a). These lines are identified to be HCP phases by DF image (Fig. 9b) and SAD pattern (Fig. 9c). Twins are not found in this specimen.

**Figure 10:**
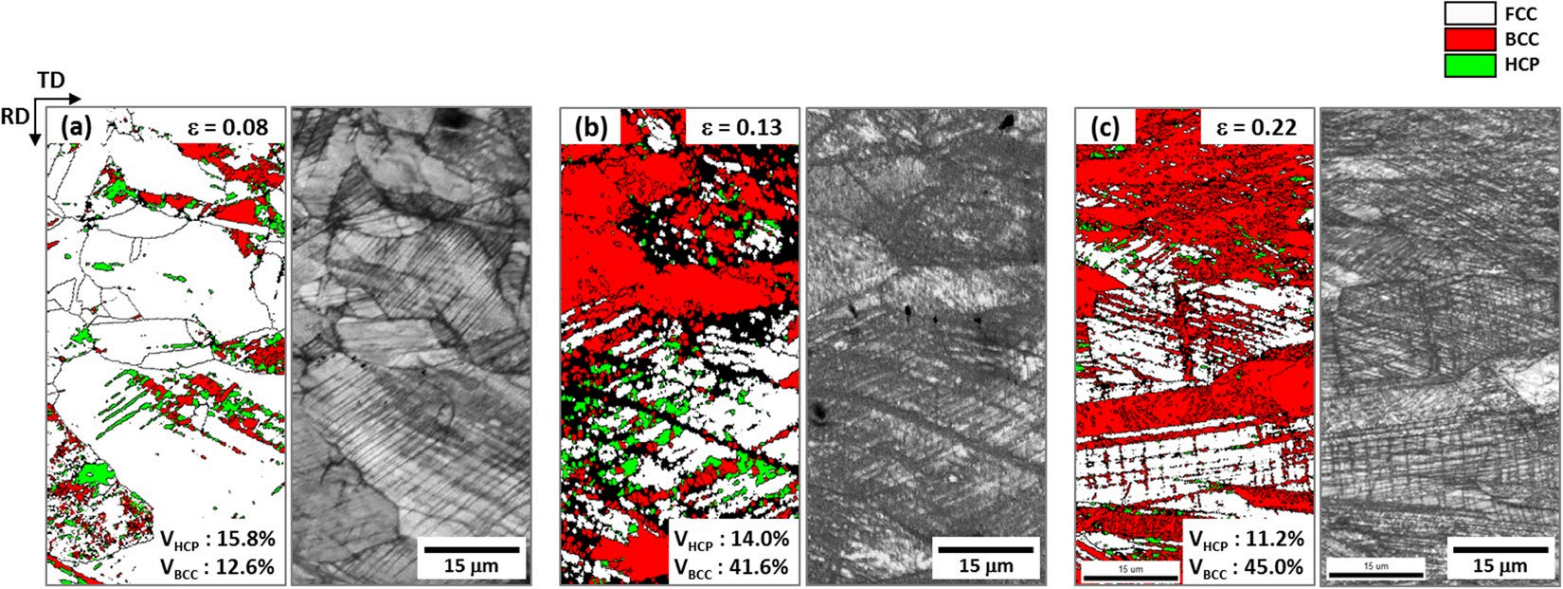
EBSD phase and IQ maps of the dynamically compressed specimen at true strains of (**a**) 0.08, (**b**) 0.13, and (**c**) 0.22. At the strain of 0.08, HCP and BCC phases as well as parallel lines are formed, and their formation becomes active as the strain increases.

**Figure 11:**
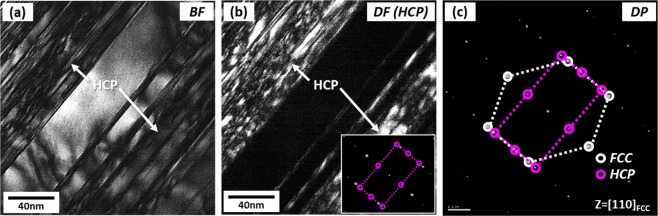
TEM (**a**) BF and (**b**) DF images and (**c**) SAD pattern of parallel lines existed inside an FCC grain of the dynamically compressed specimen at the true strain of 0.13. Many parallel lines inside the FCC grain are identified to be HCP phases.

Figure 10a–c shows EBSD phase and IQ maps of the dynamically compressed specimen at true strains of 0.08, 0.13, and 0.22. HCP and BCC phases are formed at the initial strain (0.08) (Fig. 10a), and their formation becomes active as the strain increases (Fig. 10b,c)). The overall phase formation behavior is similar to that of the quasi-statically compressed specimen (Fig. 8a–c). According to the TEM observation (Fig. 11a–c), all parallel lines are not twins but HCP phases.

**Figure 12:**
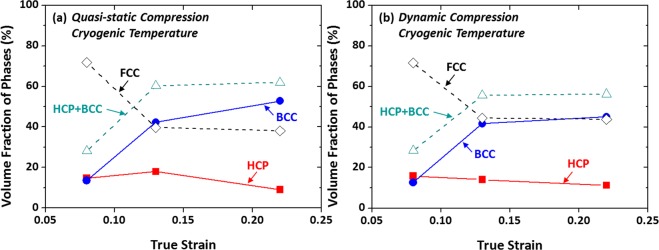
Volume fractions of FCC, BCC, and HCP phases present in the (**a**) quasi-statically and (**b**) dynamically compressed specimens as a function of compressive true strain at cryogenic temperature. Under quasi-static compression, the fraction of BCC phase increases rather abruptly as the strain increases to 0.13, while that of HCP phase increases slightly. With the further compressive strain to 0.22, the fraction of BCC phase increases, whereas that of HCP phase decreases, and thus the total (BCC + HCP) volume fraction tends to be saturated. Under dynamic compression, the variation of phase fractions is similar to that of the quasi-statically compressed specimen. Overall (BCC + HCP) volume fractions are somewhat lower in the dynamically compressed specimen.

Figure 12a,b shows variations in volume fractions of phases present in the quasi-statically and dynamically compressed specimens as a function of true strain. Under quasi-static compression, both HCP and BCC phases are formed at the strain of 0.08, and their volume fractions are about 15% (Fig. 12a). As the strain increases to 0.13, the BCC fraction rises rather abruptly, while the HCP fraction increases slightly. With the further strain to 0.22, the BCC fraction increases, while the HCP fraction decreases, and thus the total (BCC + HCP) fraction tends to be saturated. Under dynamic compression, the variation of phase fractions is similar to that under quasi-static compression (Fig. 12b), and the saturation of total (BCC + HCP) fraction also appears at the strain of 0.13 or higher. Overall (BCC + HCP) volume fractions are somewhat lower in the dynamically compressed specimen.

## Discussion

### Room-temperature deformation mechanisms: TRIP and TWIP

A viscous phonon-drag effect and a thermally-activated nucleation of dislocations generally operate at high strain rates^16,23–27^. Under the present dynamic loading case (compressive strain rate of ~3000 s^−1^) of the VCrFeCo HEA having metastable FCC matrix, however, the viscous drag effect becomes much more dominant than the thermally-activated dislocation nucleation^28^. As the dislocation movement becomes difficult under the dynamic loading, the amount of generated dislocations becomes larger at a given strain than that of the quasi-static loading case^29^. According to this strain-rate effect, the dynamic strength is higher than the quasi-static strength (Fig. 1a).

BCC-phase martensites are formed at room temperature by the FCC to BCC TRIP during quasi-static and dynamic compressive deformation (Figs 2a–d and 4a–d). Since this formation of BCC martensite is largely influenced by the enhancement of strain-rate sensitivity due to limited slip systems than the FCC phase^21^, both quasi-statically and dynamically compressed specimens show very high strain-hardening rate and consequently maximum compressive strength over 1.6 GPa (Fig. 1a,b and Supplementary Table 1). The dynamic compressive strength is much higher (by 240 MPa) than the quasi-static strength, which can be readily explained by a strain-rate-hardening effect^19,20^.

It is noted that the BCC martensite fraction is lower by about 10 vol.% after the true strain of 0.15 in the dynamically compressed specimen than in the quasi-statically compressed specimen (Fig. 6a,b). This decreased amount of BCC martensite is caused by the increase in stability of FCC phase because of the temperature rise due to adiabatic heating^30,31^. Under dynamic loading, the adiabatic heating raises the temperature and consequently the SFE or stability of FCC phase as the SFE increases with increasing temperature^32–34^. This decrease in BCC martensite lowers the strain-rate-hardening effect, and thus the difference in strength between the dynamic and quasi-static loadings becomes smaller after the strain of 0.15, as shown in Fig. 1a.

The temperature rise occurring during dynamic compressive tests was estimated to correlate the adiabatic heating and SFE increase. The temperature distribution inside the dynamically compressed specimen at room temperature was calculated by thermos-elasto-plastic simulation of finite element method (FEM) using an ABAQUS ver. 6.9 and explicit schemes of dynamic solutions because of difficulties in the experimental temperature measurement. Dynamic conditions and specimen geometries were based on experimental ones. The number of elements was 31,145 for the cylindrical 5φ × 5-mm specimen. The tool was modeled as a rigid body, and the Poisson’s ratio of 0.299 and density of 7.668 g/cm^3^ were used. Details on FEM simulations are described in references^17^.

**Figure 13:**
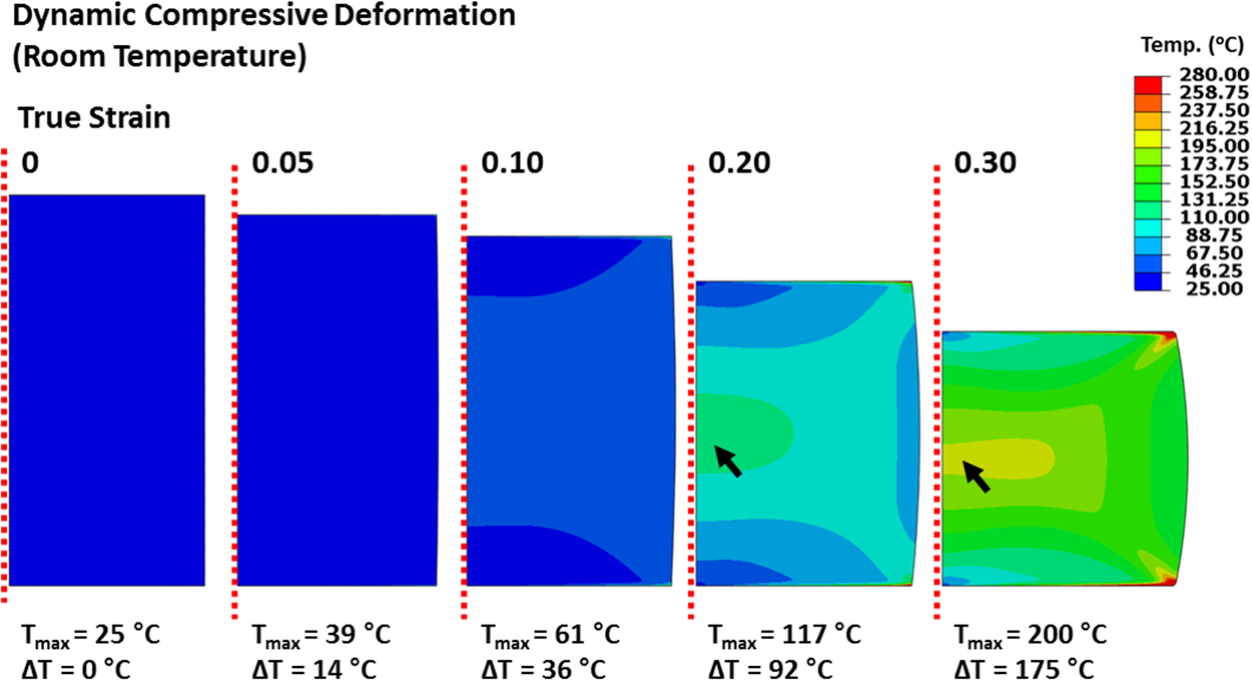
Temperature distributions calculated by finite element method (FEM) simulations at true strains 0.05, 0.1, 0.2, and 0.3 inside the dynamically compressed specimen. The temperature localization is concentrated at the specimen center, while the localization at specimen edges is released as the heat is readily emitted outside. The peak temperature of the specimen center reaches 200 °C at the true strain of 0.3.

Figure 13 shows FEM-simulated temperature distributions at true strains 0.05, 0.1, 0.2, and 0.3 inside the dynamically compressed specimen. The temperature localization occurs at specimen edges along the shear direction, and becomes more serious as the true strain increases. The temperature is concentrated at the specimen center, while its localization at specimen edges is released as the heat is readily emitted outside. The peak temperature of the specimen center reaches 200 °C at 0.3. The temperature rise of 175 °C considerably raises the SFE of FCC phase, and reduces the amount of transformed BCC martensite (Fig. 6b), thereby leading to the decrease in strengthening.

However, the compressive stress of the dynamically compressed specimen keeps increasing with a high strain-hardening rate over that of the quasi-statically compressed specimen (Fig. 1a). This implies that another deformation mechanism can work together with the BCC TRIP. According to the TEM data (Fig. 5d–f), many twins are observed in the dynamically compressed specimen. The existence of twins is associated with the increase of SFE of FCC phase due to the temperature rise, which might reach the regime of TWIP mechanism, like in the SFE-range diagram of austenitic high-Mn steels^17,35–38^. Since twins act as obstacles against dislocation movement, they induce the highly-sustained strain-hardening rate by the dynamic Hall-Petch effect^39,40^, which results in an excellent compressive strength.

### Cryogenic-temperature deformation mechanisms: TRIP

At cryogenic temperature, the stability of FCC phase contributes to mechanical response in the TRIP mechanism. For example, there exists a small stress drop in the stress-strain curve of the quasi-statically compressed specimen (a blue-arrow mark in Fig. 7a) because of a down-up-down-shaped strain hardening (Fig. 7b). It is known that this stress drop or ‘softening’ phenomenon is mainly attributed to the rapidly decreased strain-hardening rate due to the FCC to HCP transformation in the early deformation stage^41,42^. It has also been found in in ultra-fine-grained materials due to the lack of mobile dislocations^43,44^ as well as metastable austenitic stainless steels^45^, Fe-Mn alloys^46,47^, and Fe-Mn-Si alloys^48^. Gunter *et al*.^49^ proposed that the easy deformation and rapid hardening stages were closely related with ε- and α’-martensites, respectively. According to Tamura *et al*.^50^, the ε-martensite formation produced this softening in the early deformation stage of FCC-based Fe-Ni and Fe-Cr-Ni alloys. Thus, the softening would occur at low strains by the formation of HCP martensite, like in FCC-based alloys.

The microstructural evolution and phase stability at cryogenic temperature also show similar trends to those at room temperature. The EBSD data of the quasi-statically compressed specimen (Fig. 8a–c) indicate that the martensitic transformation is identified as a strain-induced one because most of BCC martensites are formed inside FCC grains, and that the amount of transformed BCC martensite is larger at cryogenic temperature. This increase in BCC martensite at cryogenic temperature is caused by the decrease in stability of FCC phase with decreasing temperature^32–34^. Thus, the cryogenic-temperature strength is higher than the room-temperature strength.

The temperature rise as well as the decrease in temperature should be considered under dynamic loading. Since the SFE of FCC phase increases with increasing temperature, it is higher in the dynamically compressed specimen, which results in the lower volume fraction of HCP and BCC martensites (Fig. 12a,b). Twins do not exist because the increased SFE of FCC phase might not be enough to reach the regime of TWIP mechanism. However, the dynamically compressed specimen shows the higher strength than the quasi-statically compressed specimen as the strain-rate-hardening effect is added with the TRIP.

Deformation mechanisms varied with SFE of FCC phase, test temperature, and loading condition are schematically illustrated in Supplementary Fig. 3. As the SFE increases, they change from TRIP to TWIP^51,52^, and the SFEs are approximately estimated on the line of SFE (red- and blue-arrow marks). The SFE of the quasi-statically compressed specimen at room temperature lies within the TRIP regime (red arrow mark) (Figs 2a–d and 3a–e). Under dynamic loading, the SFE increases to reach the regime of TWIP mechanism, and thus twins as well as HCP and BCC martensites are formed (Figs 4a–d and 5a–f). When the quasi-static loading changes to the dynamic one, deformation mechanisms shift toward the right. Considering the favorable effect of twinning on strain hardening and strengthening, thus, the higher strength in the dynamically compressed specimen containing both twins and martensites can be plausibly interpreted by Supplementary Fig. 3.

At cryogenic temperature, martensites are formed alone without twins (Fig. 8 through 11). The SFE of the dynamically compressed specimen is situated within the TRIP regime, while the SFE of the quasi-static specimen lies below that of the dynamic specimen (blue arrow marks). Thus, the TRIP amount in the dynamic specimen is smaller. However, the dynamic strength is higher as the strain-rate-hardening effect is added.

## Conclusions

Quasi-static and dynamic compressive properties of an FCC-based metastable V10Cr10Fe45Co35 HEA showing both TRIP and TWIP were investigated at room and cryogenic temperatures, and a transition of TRIP and TWIP mechanisms was examined by detailed microstructural evolutions.

1. The BCC martensite was formed by the FCC to BCC TRIP during the quasi-static and dynamic compression at room temperature, and resulted in high maximum compressive strength over 1.6 GPa together with high strain-hardening rate. The dynamic strength was higher by 240 MPa than the quasi-static strength because of strain-rate-hardening effect, and kept increasing with a high strain-hardening rate from the early deformation stage as the twinning became activated.
2. The SFE of the quasi-statically compressed specimen at room temperature was situated within the regime of TRIP. Under dynamic loading, twins as well as HCP and BCC martensites were formed as the SFE was increased by the temperature rise to reach the regime of TWIP. This indicated that deformation mechanisms were shifted from the TRIP to TWIP with increasing loading rate. Considering the favorable effect of twinning on both strain hardening and strengthening, thus, the dynamically compressed specimen containing both twins and martensites showed the higher strength than the quasi-statically compressed specimen.
3. The amount of FCC to BCC TRIP was larger at cryogenic temperature than at room temperature because of the decrease in stability of FCC phase with decreasing temperature, and thus the cryogenic-temperature strength was higher than the room-temperature one. The SFE of FCC phase was higher in the dynamically compressed specimen because of the temperature rise, which resulted in the lower volume fractions of HCP and BCC martensites. Twins did not exist because the increase in SFE of FCC phase might not be enough to reach the regime of TWIP. However, the dynamically compressed specimen showed the higher strength than the quasi-statically compressed specimen because the strain-rate-hardening effect was added with the TRIP.

## Methods

### Fabrication

The HEA (V10Cr10Fe45Co35 (at.%)) was fabricated by a vacuum-induction melting route after master alloy plates were made from pure element powders (99.9% purity at least). Fe is a cost-effective element in FCC-based HEAs, and its increased content helps to promote the formation of BCC- or HCP-phase martensite. The Fe content is set to be 45 at.%, based on an alloy-design strategy underlying that the HEA is not Fe-based alloys but a multi-principal-element material^53–55^. Cr which improves the corrosion resistance is set to be 10 at.%. As a strong bonding element with Fe, Co, and Ni, 10 at.% of V is added, if the formation of sigma phase is suppressed^56,57^. The addition of Co works for the increase in BCC martensite in FCC-based metastable HEAs.

Master alloy plates (weight; about 7 kg) was molten and poured into an Y-shaped rectangular graphite module. In order to make the HEA composition uniform, a bottom part of Y-shaped ingot was sectioned and homogenized at 1100 °C for 6 hrs. The ingot (size; 110 × 80 × 60 mm) was sand-blasted, cold-rolled (reduction ratio; 50%), annealed at 950 °C for 1 hr, and quenched.

### Microstructural analyses

Phases were identified by using X-ray diffraction (XRD), and their volume fractions were quantified by a method proposed by Moser^22^ using integrated intensities of (110)_α_, (200)_α_, (211)_α_, and (220)_α_ peaks for BCC phase, (111)_γ_, (200)_γ_, (220)_γ_, (311)_γ_, and (222)_γ_ peaks for FCC phase, and (10–10)_ε_, (10–11)_ε_, and (10–12)_ε_ peaks for HCP phase. Here, α-, γ-, and ε-phases existed in high-Mn steels are equivalent to BCC, FCC, and HCP phases, respectively, in FCC-based HEAs. Deformation mechanisms were examined by using electron back-scatter diffraction (EBSD, step size; 0.15 μm) and transmission electron microscopy (TEM). TEM thin foils were prepared by electro-polishing in a 90%-CH_3_COOH and 10%-HClO_4_ solution, and were observed by using a TEM (2100, Jeol, Japan) at 200 kV.

### Compression tests

Cylindrical specimens (diameter; 5 mm, height; 5 mm, longitudinal orientation) were quasi-statically compressed at room and cryogenic temperatures at a strain rate of 10^−3^ by using a universal testing machine (1361, Instron, USA, capacity; 100 kN). A split Hopkinson pressure bar was used for dynamic compressive tests^17^. The same-size cylindrical specimen was situated between incident and transmission bars, and was compressed by a high-speed 19-mm-diameter striker bar under an air pressure of 0.2 MPa (compressive strain rate; ~3000 s^−1^). Raw compressive curves were smoothened by an adjacent-averaging method, and entire tests were performed three times for each datum. Test details were explained in a reference^17^.

## Supplementary information

Supplementary Information

## Data Availability

The data that support the findings of this study are available from the corresponding author upon reasonable request.
