# Supplementary material for: Effects of strain rate on room- and cryogenic-temperature compressive properties in metastable V10Cr10Fe45Co35 high-entropy alloy
Source: Sci Rep. 2019 Apr 16;9:6163. doi: 10.1038/s41598-019-42704-x (PMC6467881; doi:10.1038/s41598-019-42704-x)
Supplement: Supplementary file 1 — Supplementary Information [file 41598_2019_42704_MOESM1_ESM.docx]

**Supplementary Information**

**Effects of strain rate on room- and cryogenic-temperature compressive properties**

**in metastable V10Cr10Fe45Co35 high-entropy alloy**

**Hyejin Song, Dong Geun Kim, Dae Woong Kim, Min Cheol Jo, Yong Hee Jo,**

**Wooyeol Kim, Hyoung Seop Kim, Byeong-Joo Lee, Sunghak Lee^*^**

***Center for High Entropy Alloys***

***Pohang University of Science and Technology, Pohang 790-784, Korea***

*Corresponding author: Sunghak Lee

[shlee@postech.ac.kr](mailto:shlee@postech.ac.kr)

Tel: +82-54-279-2140

Fax: +82-54-279-2399

**Supplementary Figures**


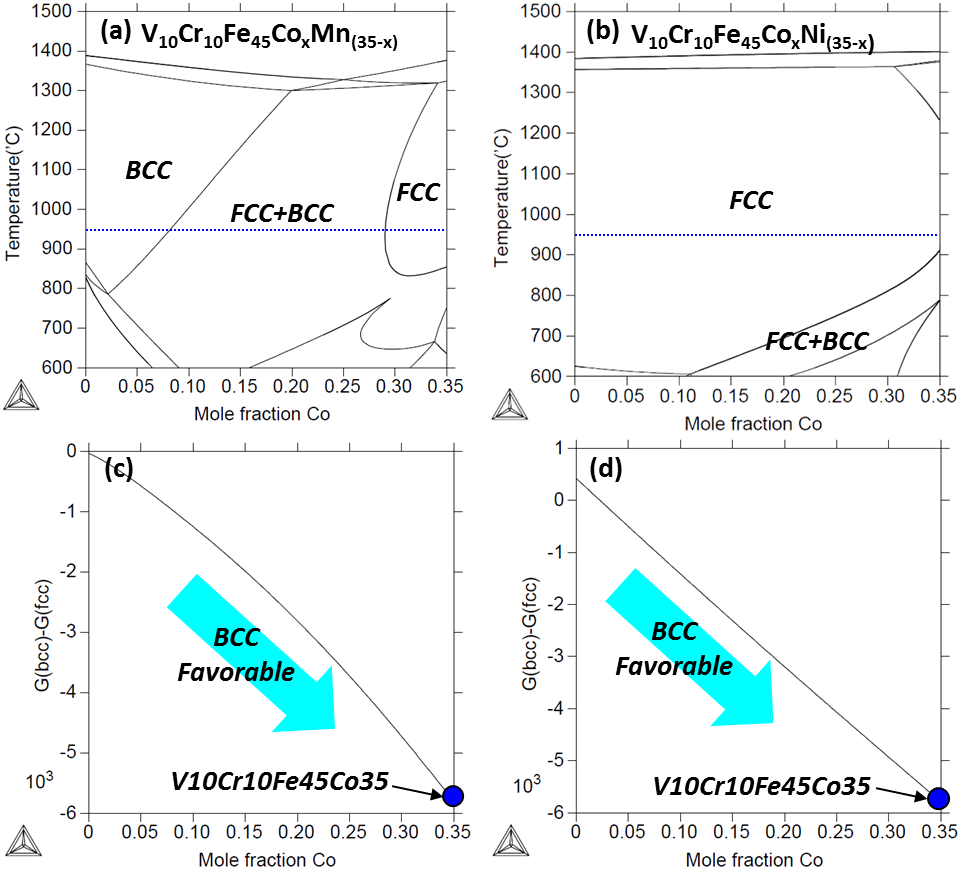


Supplementary Figure 1. (a,b) Equilibrium phase diagrams in the temperature range of 600 °C~1500 °C and (c,d) differences in Gibbs free energy between BCC and FCC phases (ΔG^FCC→BCC^, stability of BCC phase) of Fe_45_Cr_10_V_10_Co_x_Mn_(35-x)_ and Fe_45_Cr_10_V_10_Co_x_Ni_(35-x)_ systems. In order to obtain the stable single FCC phase at 950 °C (HEA annealing temperature, dotted lines in (a,b)) and to generate the maximal transformation from FCC to BCC, the Co content is set to be 35 at.% (HEA composition; V10Cr10Fe45Co35 (at.%)) in this study.


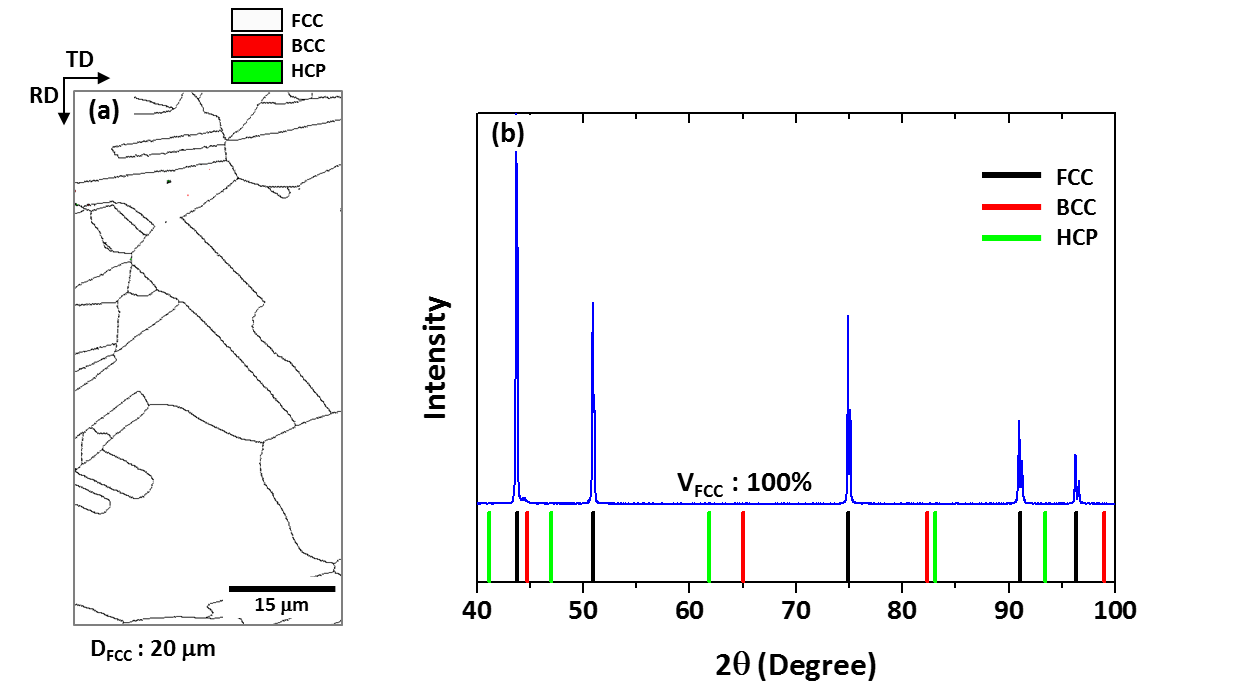


Supplementary Figure 2. (a) EBSD phase map and (b) XRD data of the V10Cr10Fe45Co35 HEA. The HEA shows a single FCC phase, which satisfies the first objective of the present HEA design (a single FCC phase in the as-annealed state), and its average FCC grain size is 20 μm.


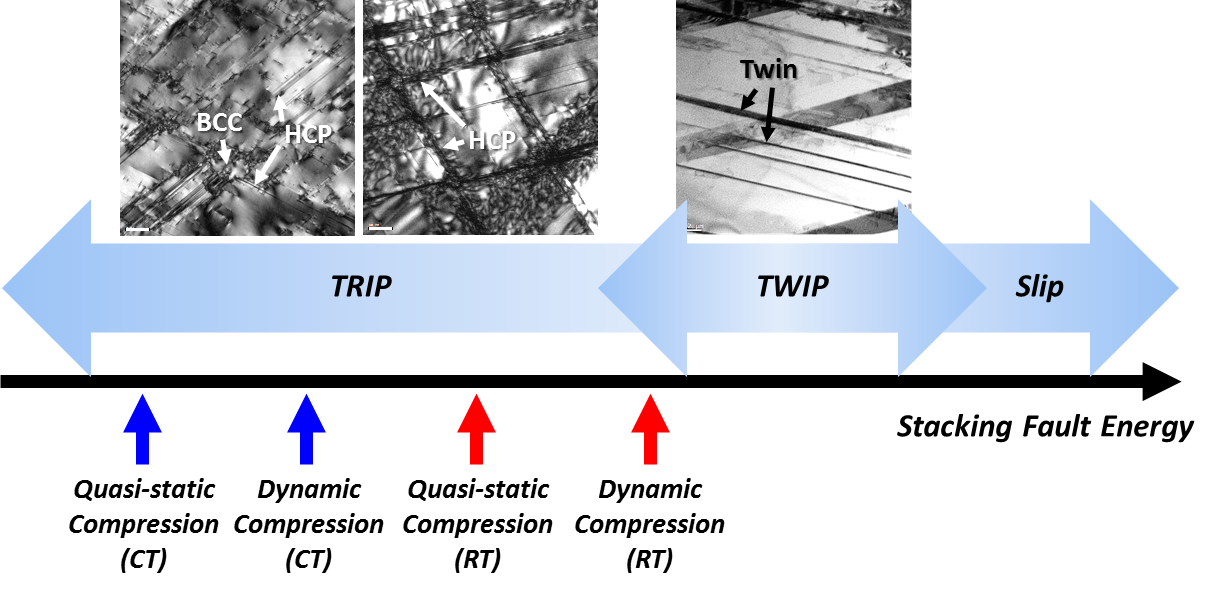


Supplementary Figure 3. Schematic diagram of deformation mechanisms varied with SFE of FCC phase, loading condition, and test temperature. Deformation mechanisms are changed from TRIP to TWIP as the SFE increases, and SFEs are estimated as marked by red and blue arrows on the SFE line. Based on the microstructural evolution, the SFE of the quasi-statically compressed specimen at room temperature is situated within the deformation mechanism of TRIP. Deformation mechanisms are shifted to the right side when the loading condition is changed from quasi-static one to dynamic one. At cryogenic temperature, the SFE of the dynamically compressed specimen is situated within the deformation mechanism of TRIP, while the SFE of the quasi-statically compressed specimen lies below that of the dynamically compressed specimen.

**Supplementary Tables**

**Supplementary Table 1.** Room- and cryogenic-temperature compressive test results of the V10Cr10Fe45Co35 (at.%)) HEA specimens.

| Test Temperature | Compressive Loading Condition | Yield Compressive Strength (MPa) | Maximum Compressive Strength (MPa) | Total  Strain  (%) |
| --- | --- | --- | --- | --- |
| Room  Temperature | Quasi-static | 364 ± 10 | 1682 ± 17 | 26.2 ± 1.3 |
|  | Dynamic | 640 ± 15 | 1925 ± 12 | 25.9 ± 1.1 |
| Cryogenic  Temperature | Quasi-static | 534 ± 17 | 1735 ± 11 | 21.2 ± 1.2 |
|  | Dynamic | 1083 ± 20 | 2245 ± 10 | 21.6 ± 1.1 |
